# Pseudo dilated cardiomyopathy: Dilated cardiomyopathy‐like changes due to a combination of stuck mechanical mitral valve and coronary microvascular dysfunction—An autopsy case

**DOI:** 10.1002/ccr3.8136

**Published:** 2023-11-14

**Authors:** Sato Namura, Hiroki Matsuzoe, Mayumi Inaba, Ryo Nishio, Daisuke Matsumoto, Hiroshi Takaishi

**Affiliations:** ^1^ Department of Cardiovascular Medicine Yodogawa Christian Hospital Osaka Japan; ^2^ Department of Pathology Yodogawa Christian Hospital Osaka Japan

**Keywords:** biventricular systolic dysfunction, mitral valve replacement, mural thrombi, myocardial infarction with non‐obstructive coronary arteries, non‐ischemic dilated cardiomyopathy, stuck valve

## Abstract

Thrombus formation in the microvessels and endocardium was suggestive of endothelial cell damage, myocardial ischemia, and a decreased coronary flow reserve. Sustained pulmonary hypertension due to thrombosis worsened the biventricular dysfunction.

## 1 CASE PRESENTATION

**FIGURE 1:**
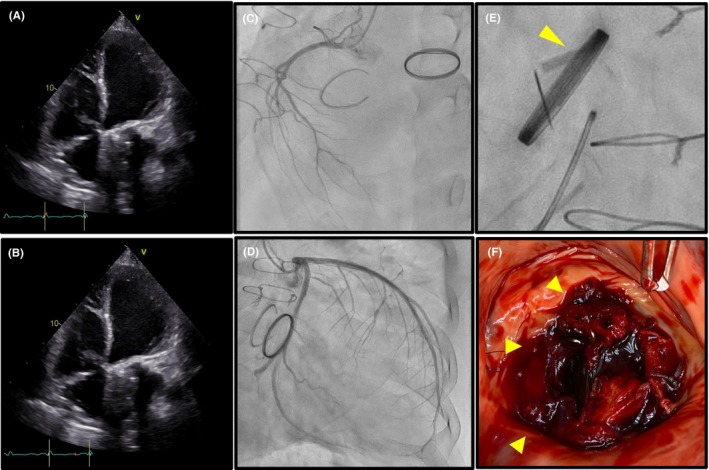
(A, B) Transthoracic echocardiography shows severe biventricular dysfunction (A: end‐diastolic, B: end‐systolic). (C, D) Coronary angiography reveals normal coronary flow (C: right coronary artery, D: left coronary artery). (E) The lateral leaflet of the mechanical valve is dysfunctional and nonmobile (yellow arrowhead). (F) Autopsy findings reveal an organized thrombus stacked in the lateral leaflet (yellow arrowhead), while the mobility of the medial leaflet is unaffected.

A 52‐year‐old man who had previously undergone a total chordal sparing mitral valve replacement with a 29 mm mechanical heart valve and a tricuspid annuloplasty with a 28 mm annuloplasty ring for infective endocarditis at 45 years of age was referred to our hospital. The patient had stopped his hospital visits and his prescribed warfarin. Although the left ventricular ejection fraction on the sixth postoperative day was preserved, transthoracic echocardiography revealed diffuse severe hypokinesis in both ventricles with left ventricular ejection fraction of 15% (Figure 1A,B) and a stuck lateral leaflet of the mechanical valve without significant mitral regurgitation (Figure 1E). Furthermore, the mean diastolic pressure gradient of the mitral prosthesis was 18.2 mmHg. Coronary angiography was normal (Figure 1C,D), and laboratory data revealed no eosinophilia and no presence of antiphospholipid antibodies. Electrocardiography revealed a normal sinus rhythm with 122 bpm, QS pattern in leads V1–V4, and only inverted T waves in leads V5–V6. However, no ST depression or elevation were observed.

**FIGURE 2:**
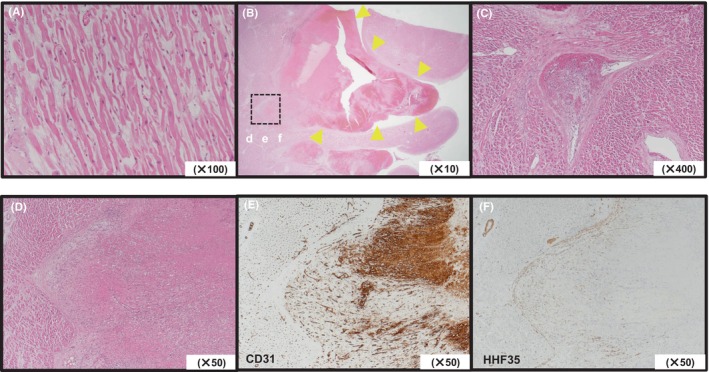
(A) Atrophied myocardial cells are present and necrotic changes are observed; however, no inflammatory cell infiltrate in the myocardium or edematous changes can be seen (×100). (B) Multifocal organizing thrombi with nuclear debris are found in both ventricles (yellow arrowhead; ×10). (C) Thrombus formation is observed in the microvasculature, along with perivascular fibrosis and mild inflammatory cell infiltration around the thrombus (×400). (D–F) Magnified microscopy images and immunostaining. (D) Thrombus formation (×50). (E, F) CD31 (endothelial cell marker) and HHF35 (muscle and muscle‐derived cell marker) staining demonstrates shedding of endothelial cells, compensatory fibrotic thickening, and perivascular fibrotic thickening (×50).

Owing to the high surgical risk (Euro SCORE II of 39.81%), we attempted thrombolysis therapy. However, sudden onset of respiratory distress and conjugate deviation led to an immediate cardiac arrest. The patient was unresponsive to subsequent cardiopulmonary resuscitation. We concluded that the patient experienced sudden cardiac death due to cardiogenic shock on the fourth day. Autopsy findings revealed an organized thrombus stacked only at the lateral leaflet (Figure 1F). Mural thrombi were found in both ventricles. Moreover, thrombi had formed in the microvasculature. There was no bacterial aggregation or deformation of the cardiomyocytes or the myocardial interstitium. Endothelial cell shedding, compensatory fibrotic thickening, and perivascular fibrotic thickening were observed. Interestingly, there were no pathological thromboembolic events in other organs and plaques in the epicardial coronary arteries (Figure 2).

## 2 DISCUSSION

This case was characterized by the absence of stenosis and plaques in the epicardial coronary arteries; however, chronic thrombotic occlusion was present at the microvascular level of the coronary arteries. Furthermore, histopathological evidence of thromboembolisms in any other organ or thrombogenic predisposition factors, such as antiphospholipid syndrome, was absent. In microvascular coronary thrombi, myocardial infarction with nonobstructive coronary arteries (MINOCA) should be considered. It is clinically heterogeneous and reportedly caused by several different clinical entities, which include coronary embolism, coronary microvascular spasm, myocarditis, and Takotsubo syndrome in cases wherein epicardial coronary artery disorders are absent. The most probable explanation in the present case is a coronary embolism.^1^

A previous systematic review of 28 studies on MINOCA reported that 14% of the study cohorts showed a tendency for thrombus formation and concluded that increased coagulability is involved in the development of MINOCA.^2^ Although the repeated systemic embolization from the mitral valve thrombus had not been pathologically confirmed, it must be pathologically considered that the thrombi found in the microvasculature originated from the thrombus attached to the leaflet of the mitral prosthesis valve. Part of the cause of death in this patient would be coagulability problems; moreover, endothelial cell loss and vascular smooth muscle cell proliferation were observed in the myocardial microvessels. We concluded that thrombus formation in endocardial microvessels caused myocardial ischemia and decreased the coronary flow reserve, leading to a deterioration of left ventricular dysfunction. Furthermore, endocardial thrombus formation was stimulated by endothelial cell damage and the absence of anticoagulation therapy. Thrombosed valves caused pulmonary hypertension leading to right ventricular dysfunction. These events caused dilated cardiomyopathy‐like changes.

## AUTHOR CONTRIBUTIONS

**Sato Namura:** Conceptualization; data curation; writing – original draft. **Hiroki Matsuzoe:** Conceptualization; data curation; formal analysis; writing – original draft; writing – review and editing. **Mayumi Inaba:** Formal analysis; funding acquisition; visualization; writing – original draft. **Ryo Nishio:** Writing – review and editing. **Daisuke Matsumoto:** Validation; writing – review and editing. **Hiroshi Takaishi:** Supervision; writing – review and editing.

## FUNDING INFORMATION

None.

## CONFLICT OF INTEREST STATEMENT

None.

## CONSENT

Written consent has been obtained from the patient during his hospitalization and his family in line with COPE guidance for the submission and publication of this imaging case and associated text.

## Data Availability

Data sharing not applicable to this article as no datasets were generated or analysed during the current study.
